# Supplementary material for: Probiotics as Anti-Tumor Agents: Insights from Female Tumor Cell Culture Studies
Source: Biomolecules. 2025 May 2;15(5):657. doi: 10.3390/biom15050657 (PMC12108976; doi:10.3390/biom15050657)

pP53 OVCAR3

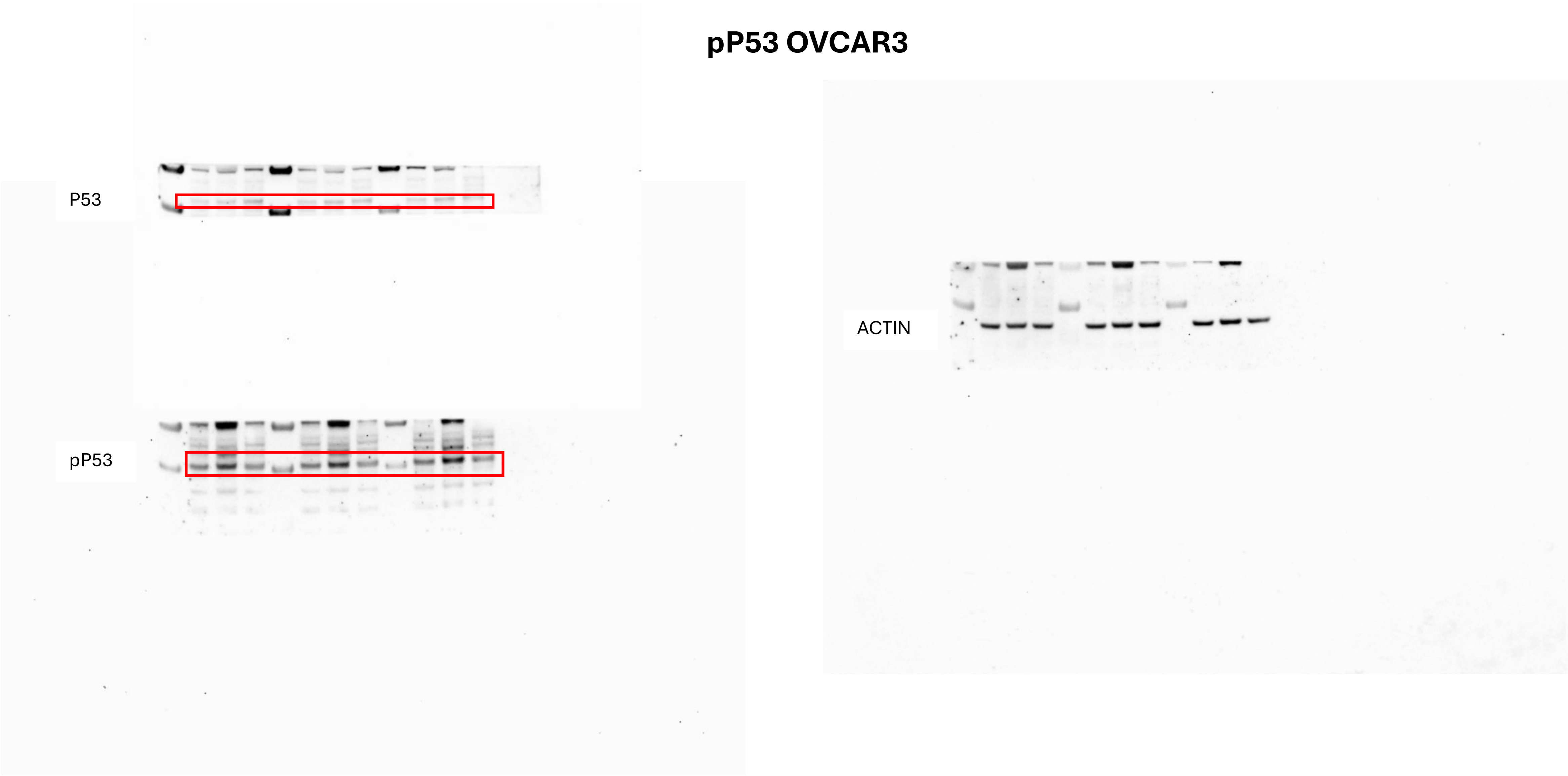

pAKT OVCAR3

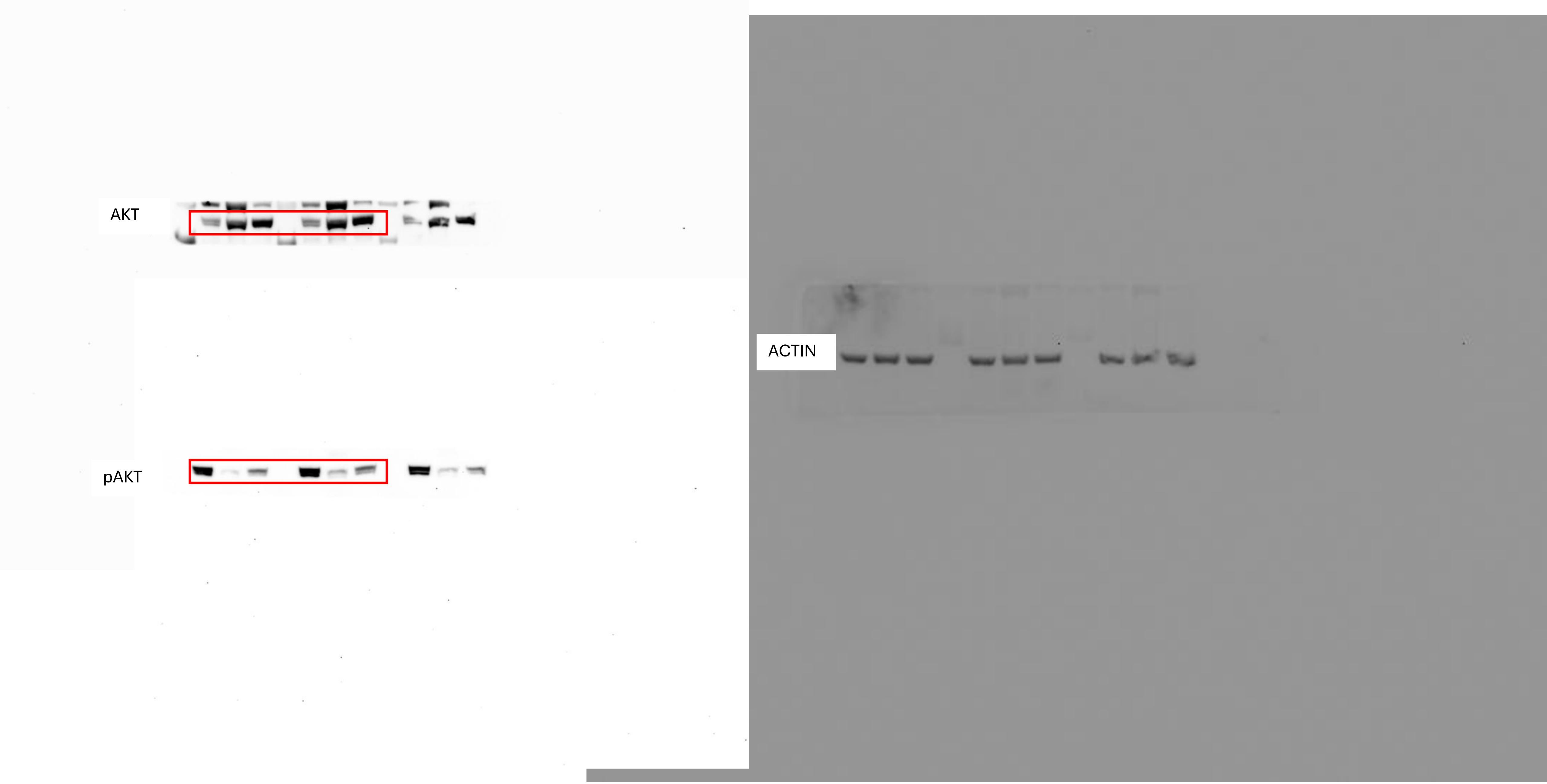

pERK1/2 OVCAR3

pERK1/2

ERK1/2

ACTIN

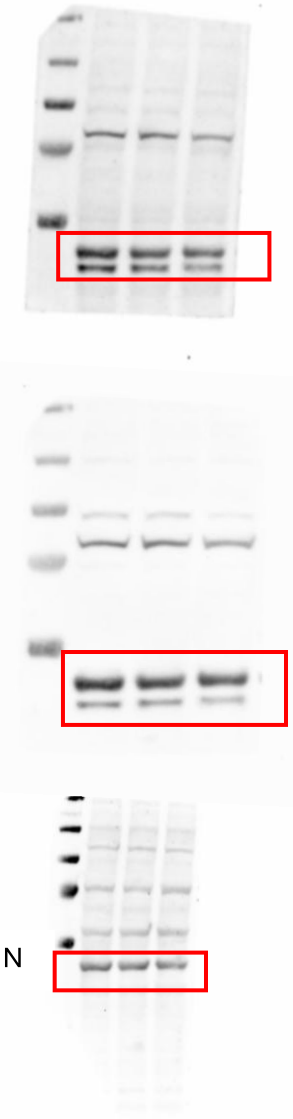

pERK1/2

ERK1/2

ACTIN

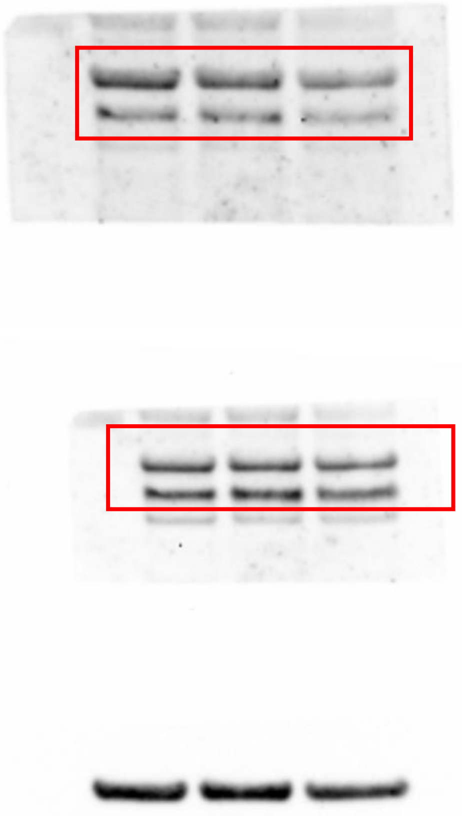

Cyclin D1 OVCAR3

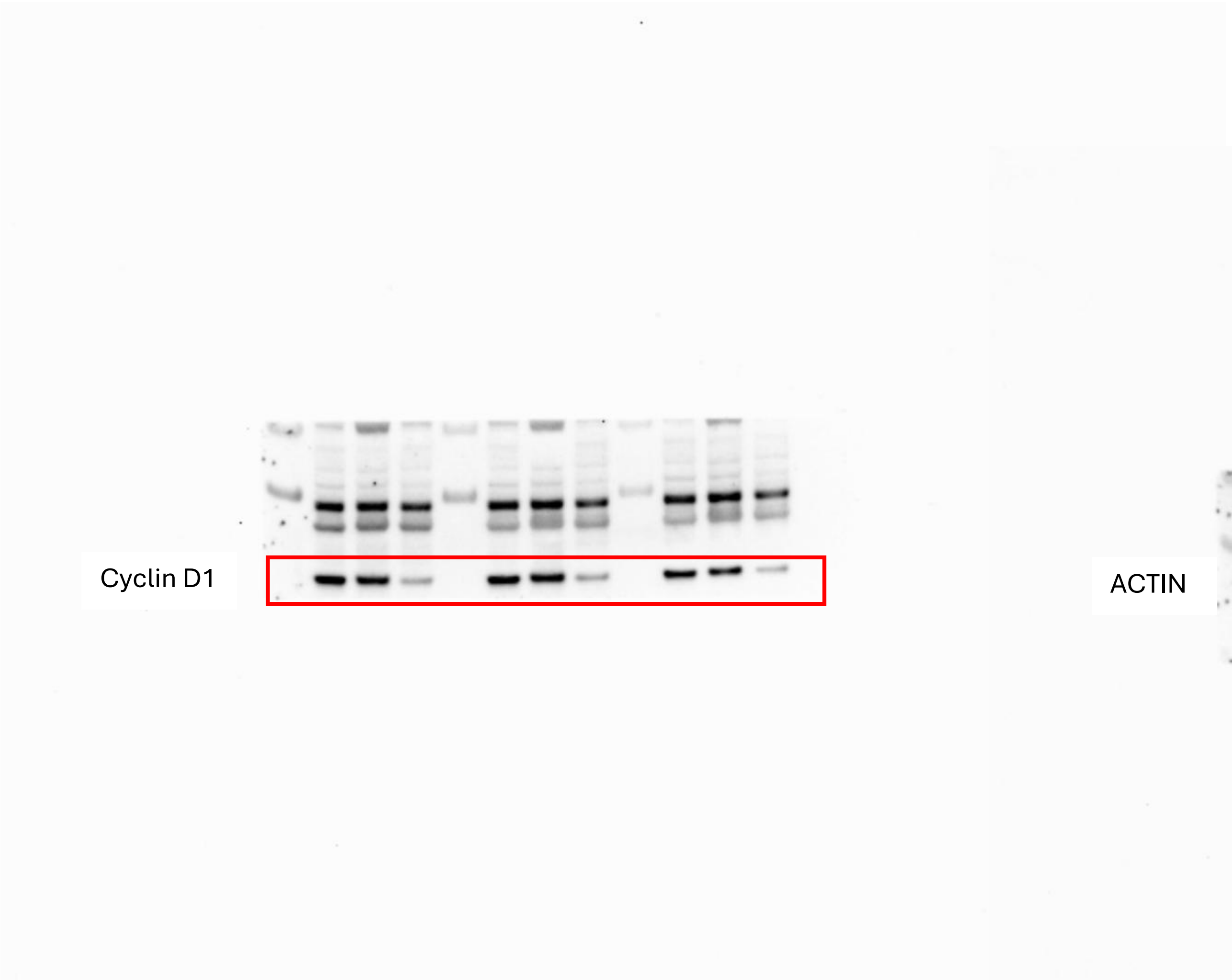

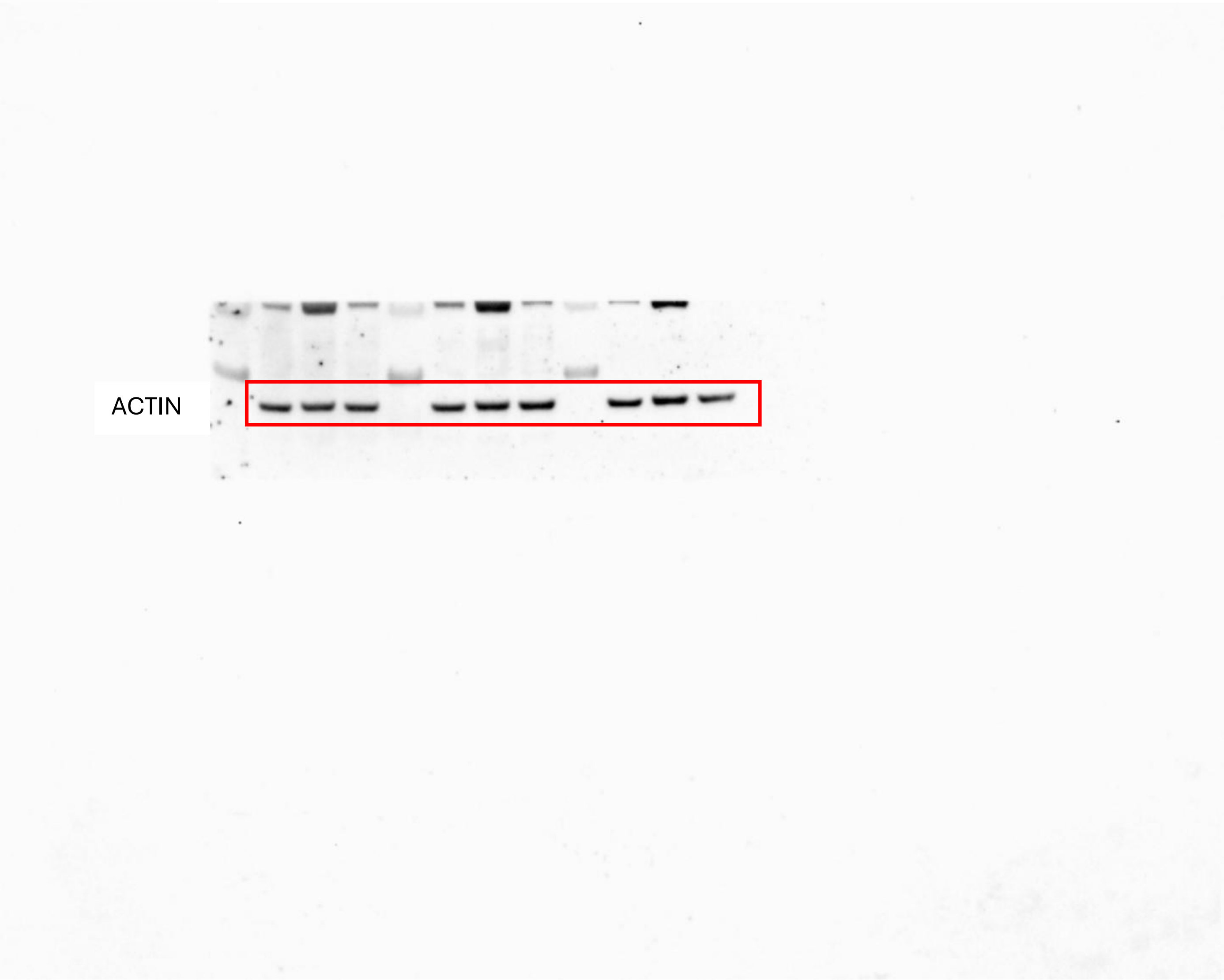

RhoA OVCAR3

RhoA

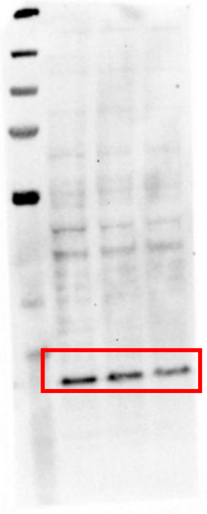

RhoA

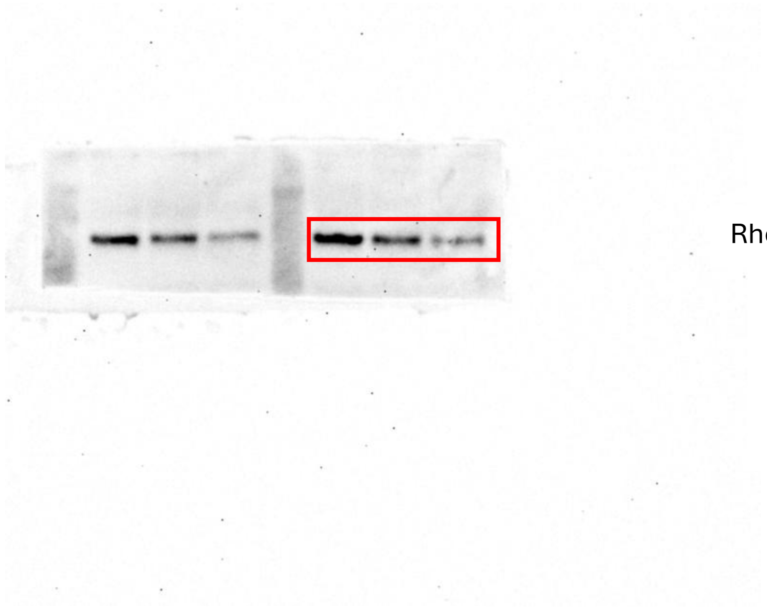

RhoA

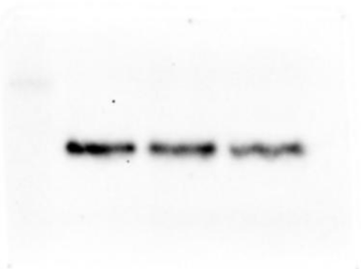

ACTIN

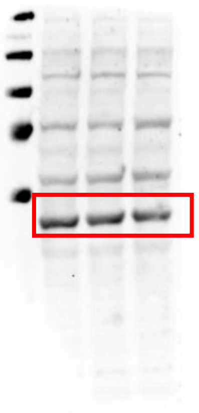

ACTIN

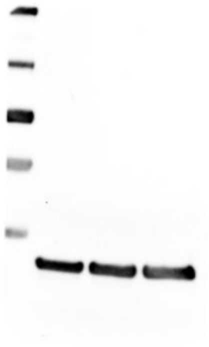

ACTIN

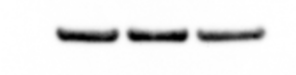

pP53 MDA-MB-231

pP53

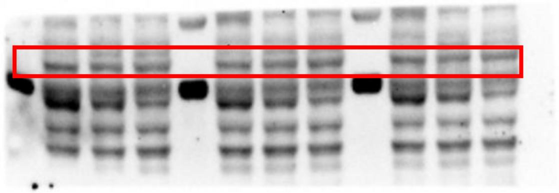

P53

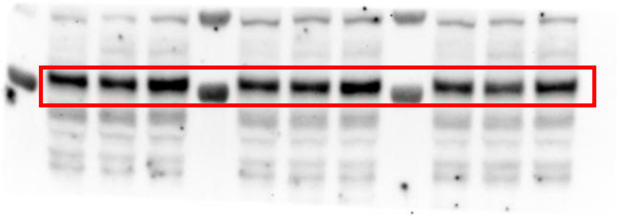

ACTIN

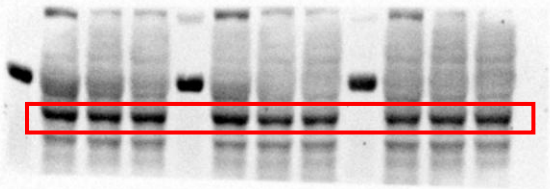

pAKT MDA-MB-231

pAKT

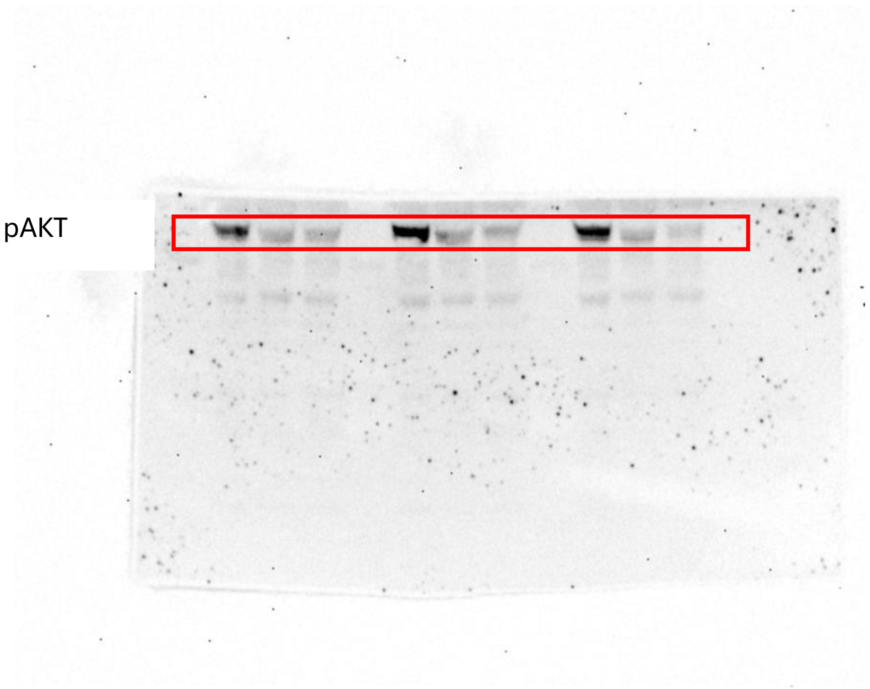

AKT

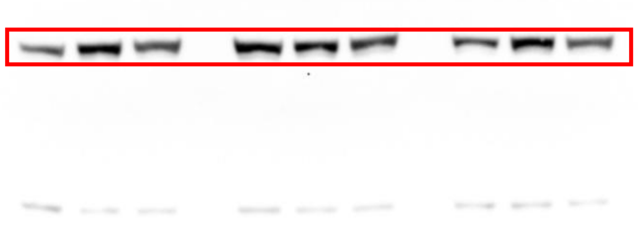

ACTIN

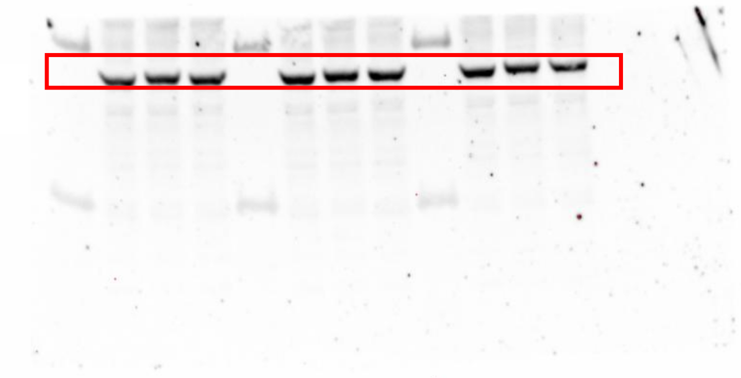

pERK1/2 MDA-MB-231

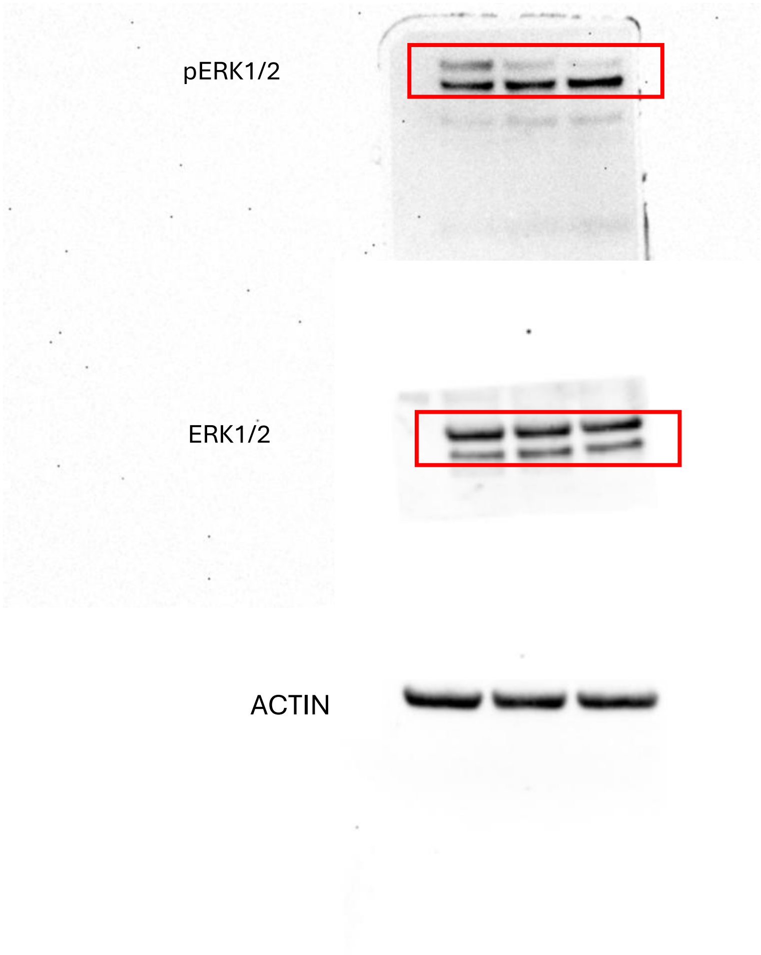

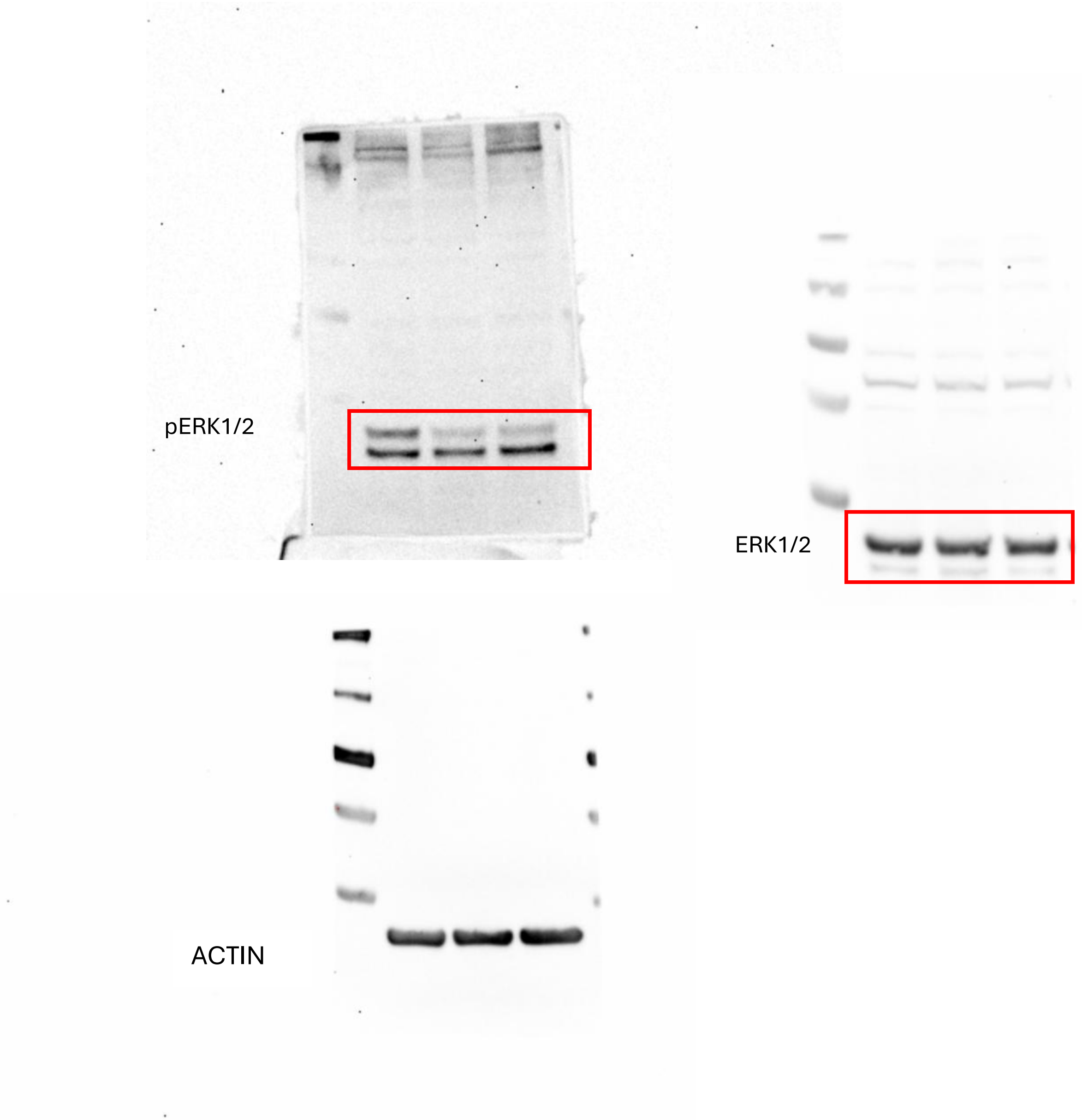

Cyclin D1 MDA-MB-231

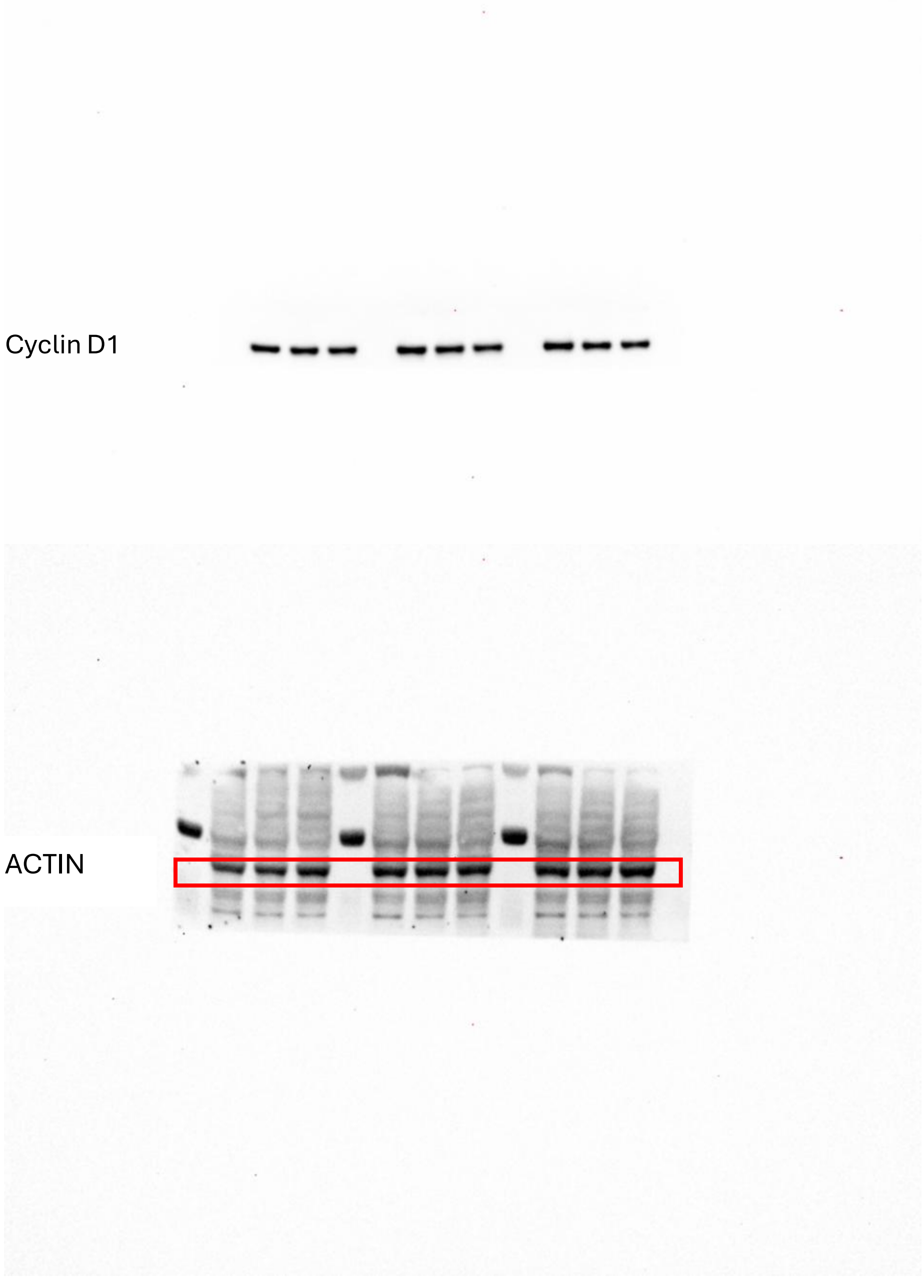

RhoA MDA-MB-231

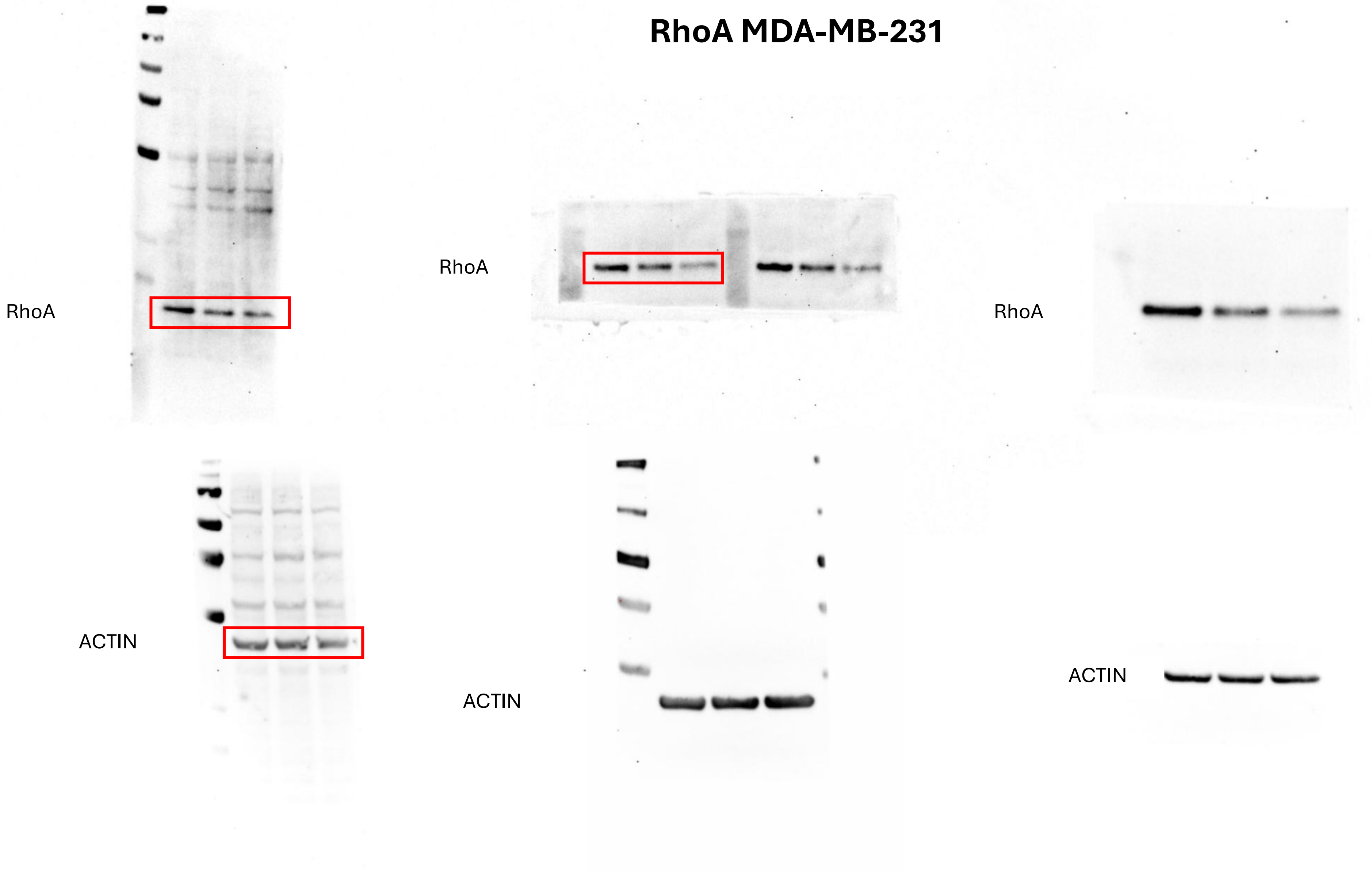

Supplement: Supplementary file 1 [file biomolecules-15-00657-s001.zip › biomolecules-3544517-Figure S2.pdf]
