# Supplementary figures and images for: Probiotics as Anti-Tumor Agents: Insights from Female Tumor Cell Culture Studies
Source: Biomolecules. 2025 May 2;15(5):657. doi: 10.3390/biom15050657 (PMC12108976; doi:10.3390/biom15050657)

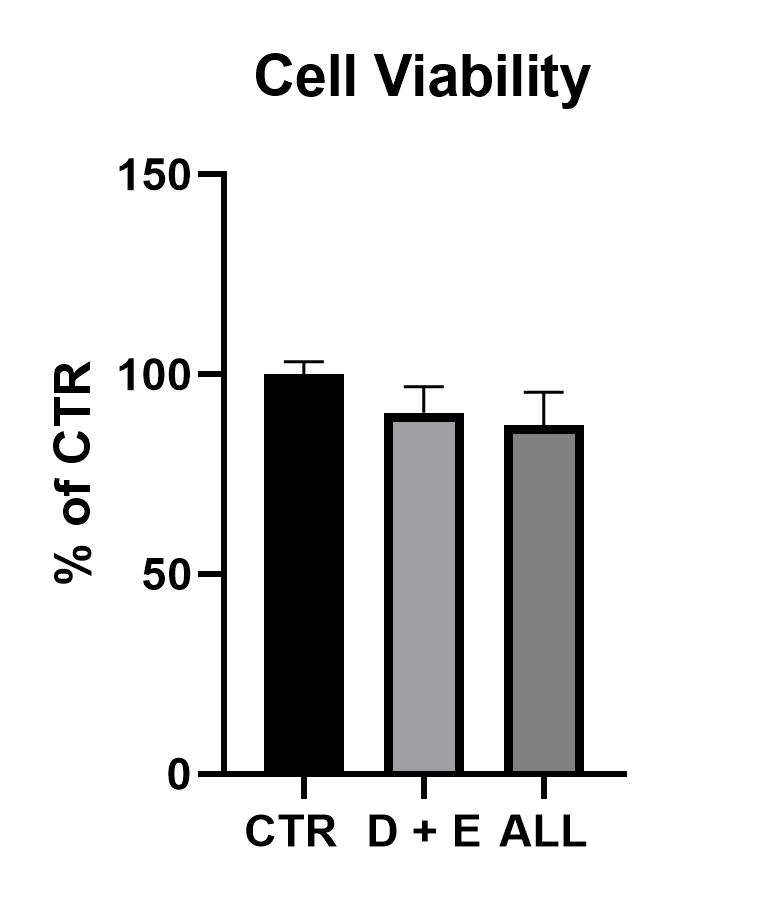

Supplement: Supplementary file 1 [file biomolecules-15-00657-s001.zip › biomolecules-3544517-Figure S1.tif]
